# Supplementary material for: Development of a Strategy for Enhancing the Biomass Growth and Lipid Accumulation of Chlorella sp. UJ-3 Using Magnetic Fe3O4 Nanoparticles
Source: Nanomaterials (Basel). 2021 Oct 22;11(11):2802. doi: 10.3390/nano11112802 (PMC8624931; doi:10.3390/nano11112802)
Supplement: Supplementary file 1 [file nanomaterials-11-02802-s001.zip › nanomaterials-1383306-supplementary.pdf]

Supplementary materials

Development of a Strategy for Enhancing the Biomass Growth and Lipid Accumulation of *Chlorella sp*. UJ-3 Using Magnetic Fe_3O_4 Nanoparticles

Feng Wang [1,*], Tingting Liu [1], Wen Guan [1], Ling Xu [1], Shuhao Huo [1], Anzou Ma [2,*], Guoqiang Zhuang [2] and Norman Terry [3]

[1] School of Food and Biological Engineering, Jiangsu University, Zhenjiang 212013, China; 2221918053@stmail.ujs.edu.cn (T.L.); ghwconan@163.com (W.G.); lxu@ujs.edu.cn (L.X.); huo@ujs.edu.cn (S.H.)
[2] Key Laboratory of Environmental Biotechnology, Research Center for Eco-Environmental Sciences, Chinese Academy of Sciences, Beijing 100085, China; gqzhuang@rcees.ac.cn
[3] Department of Plant and Microbial Biology, University of California, Berkeley, CA 94720, USA; nterry@berkeley.edu
* Correspondence: fengwang@ujs.edu.cn (F.W.); azma@rcees.ac.cn (A.M.); Tel: +86-511-887-80201 (F.W.); +86-106-292-3562 (A.M.)

1. Methods

1.1. Determination of Dissolved Fe in Culture Medium

The microalgae culture was centrifugated at 10,000 rpm for 10 min, and the supernatant was collected and dried by a lyophilizer (FreeZone®, LABCONCO, Waltham, MA USA). The dried powders were digested and then analyzed by using ICP-OES (Optima 5300DV, Pekin-Elmer, Greenville, SC, USA) according to the procedure described by John et al [1].

1.2. Fe Recovery Rate of Fe_3O_4 Nanoparticles

The microalgae culture was centrifugated at 10,000 rpm for 10 min, and the sediment was collected and washed with water for three times. The Fe_3O_4 nanoparticles in sediments were dissolved by HCl and the microalgal cells were separated by micro-filtration [2]. The resulted filtrate was dried by a lyophilizer (FreeZone®, LABCONCO, Waltham, MA, USA) and the Fe content was analyzed by using ICP-OES. The Fe recovery rate of Fe_3O_4 nanoparticles was calculated using the following equation:

$$\text{Fe recovery rate} = \text{Total Fe in the sediments} / \text{Total Fe of the added } Fe_3O_4 \text{ NPs} \times 100\%$$

The algal culture without treatment was mixed with the same dosage of Fe_3O_4 nanoparticles for 5 min and then centrifugated at 10,000 rpm for 10 min. The sediment was collected and its Fe content was analyzed. The Fe recovery rate of Fe_3O_4 nanoparticles was also calculated, which was used as the control without culture.

2. Results

2.1. Change of Dissolved Fe in the Culture Medium

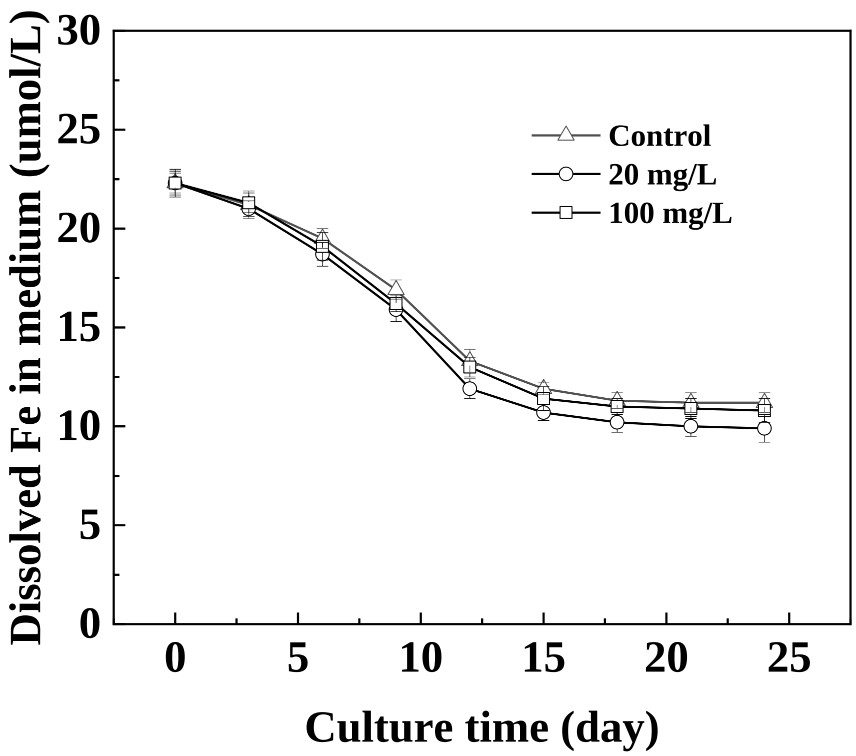

Figure S1. Change of dissolved Fe in the culture medium.

2.2. Fe Recovery Rate of Fe_3O_4 Nanoparticles

Table S1. Fe recovery rate of Fe_3O_4 NPs after culture with the algal cells for 21 days.

Concentration of Fe_3O_4 NPs (mg/L)	Fe recovery rate (%)	
	With culture	Control without culture
20	96.3±3.8	96.7±3.5
100	97.4±2.7	97.3±2.5

2.3. Change of Specific Growth Rate of Chlorella sp. UJ-3 cells during the Culture

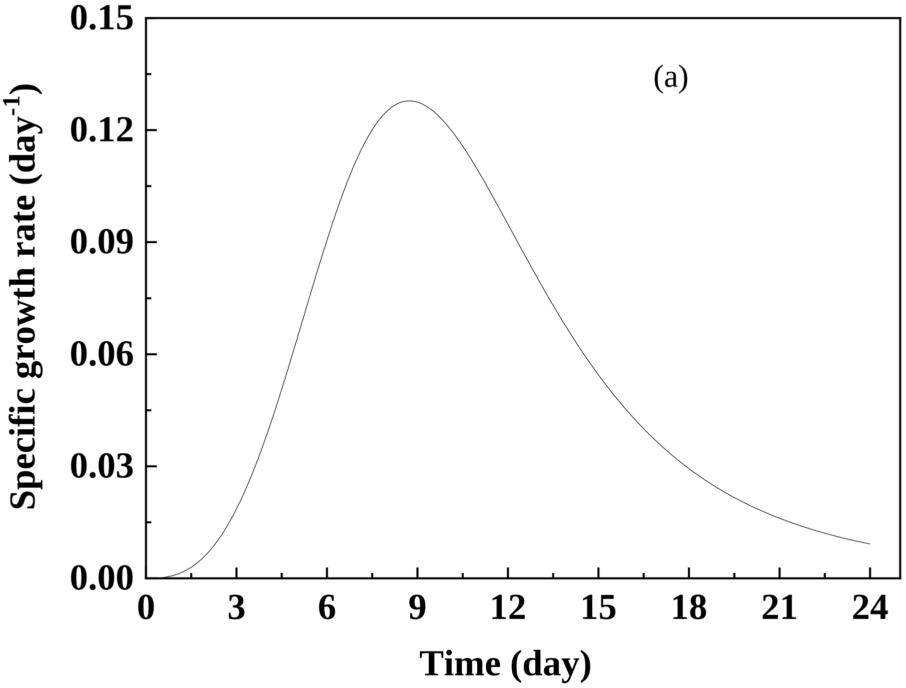

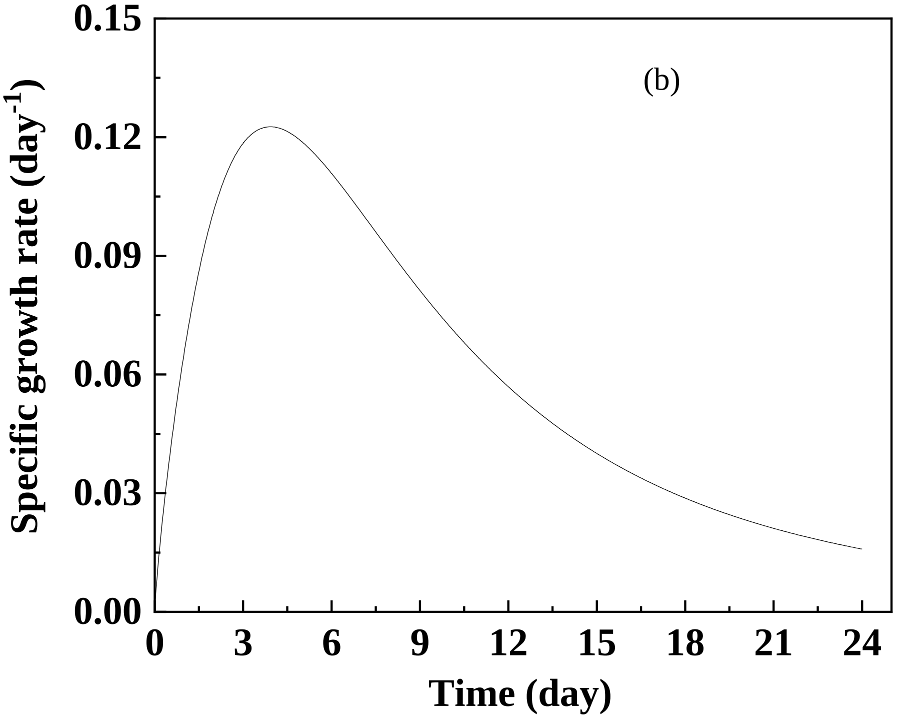

Figure S2. The specific growth rate of *Chlorella* sp. UJ-3 cells without treatment (a) and with the treatment at 100 mg/L of NPs (b).

2.4. Fatty Acid Contents and Compositions of Chlorella sp. UJ-3 Exposed to Different Concentrations of Fe_3O_4 NPs

Table S2. Fatty acid contents and compositions of *Chlorella* sp. UJ-3 exposed to different concentrations of Fe_3O_4 NPs.

Fatty acids	NPs concentration (mg/L)													
	0		10		30		50		80		100		200	
	Content (mg/gDW)	Composition (%TFA)	Content (mg/gDW)	Composition (%TFA)	Content (mg/gDW)	Composition (%TFA)	Content (mg/gDW)	Compositio n (%TFA)	Content (mg/gDW)	Composition (%TFA)	Content (mg/gDW)	Composition (%TFA)	Content (mg/gDW)	Composition (%TFA)
C12:0	1.94±0.07	1.22±0.01	2.28±0.31	1.38±0.28	3.36±0.51	2.02±0.20	3.11±0.11	1.68±0.23	4.12±0.26	1.90±0.17	4.20±0.25	1.79±0.10	1.20±0.15	0.65±0.05
C14:0	1.29±0.09	0.81±0.04	0.90±0.09	0.55±0.03	0.87±0.05	0.52±0.03	0.94±0.07	0.51±0.03	1.12±0.15	0.51±0.04	1.65±0.15	0.70±0.02	0.85±0.05	0.46±0.01
C16:0	54.53±2.02	34.49±1.45	57.20±1.53	34.67±0.85	57.49±2.04	34.50±0.98	64.19±0.98	34.57±1.12	74.05±1.84	34.15±1.65	77.05±3.3	32.77±1.78	67.39±1.30	36.26±0.76
C16:1	3.32±0.11	2.10±0.10	2.61±0.08	1.58±0.15	2.01±0.23	1.20±0.11	3.79±0.27	2.04±0.24	6.44±0.50	2.97±0.34	6.68±0.95	2.84±0.43	5.00±0.25	2.69±0.18
C18:0	7.43±0.27	4.70±0.22	8.45±0.89	5.12±0.13	8.56±0.45	5.14±0.25	11.10±0.57	5.98±0.39	12.73±1.01	5.87±0.58	16.59±0.61	7.05±0.32	12.54±0.50	6.74±0.45
C18:1	43.22±1.01	27.33±0.28	44.57±1.20	27.02±1.25	42.92±0.98	25.75±1.55	43.24±1.68	23.29±0.77	42.69±1.50	19.69±0.45	42.98±1.09	18.28±0.45	32.29±1.51	17.37±0.79
C18:2	6.70±0.36	4.24±0.32	5.08±0.57	3.08±0.41	4.93±0.76	2.96±0.55	5.93±0.42	3.20±0.37	8.55±0.50	3.95±0.12	9.59±1.00	4.08±0.67	8.14±0.48	4.38±0.22
C18:3n6	2.47±0.24	1.56±0.22	3.12±0.50	1.89±0.46	4.31±0.69	2.59±0.17	4.90±0.54	2.64±0.22	7.78±1.01	3.59±0.69	9.08±1.01	3.86±0.89	9.22±0.41	4.96±0.39
C18:3n3	27.61±1.13	17.46±0.78	29.56±1.67	17.92±1.28	30.00±1.54	18.00±1.08	33.56±1.51	18.08±1.44	39.89±2.46	18.39±0.34	44.36±2.11	18.86±1.39	30.08±1.18	16.18±0.52
C20:0	0.26±0.05	0.16±0.02	0.23±0.03	0.14±0.03	0.27±0.03	0.16±0.02	0.28±0.02	0.15±0.02	0.51±0.06	0.24±0.02	0.72±0.05	0.31±0.02	0.25±0.03	0.13±0.01
C20:1	0.80±0.11	0.51±0.06	1.53±0.10	0.93±0.14	2.07±0.14	1.24±0.05	2.32±0.11	1.25±0.08	1.99±0.57	0.92±0.45	2.09±0.16	0.89±0.06	1.45±0.28	0.78±0.02
C20:5	7.77±0.90	4.91±0.47	8.76±0.55	5.31±0.67	9.07±0.44	5.44±0.35	11.37±0.66	6.12±0.57	15.54±1.09	7.17±0.87	18.18±1.19	7.73±0.87	16.59±0.80	8.93±0.78
C22:0	0.79±0.09	0.50±0.07	0.69±0.05	0.42±0.02	0.78±0.10	0.47±0.05	0.94±0.05	0.51±0.02	1.44±0.05	0.66±0.04	1.98±0.28	0.84±0.03	0.87±0.06	0.47±0.02
Total	158.13±2.58	100.00	164.98±3.46	100.00	166.66±1.72	100.00	185.67±5.88	100.00	216.84±3.20	100.00	235.17±4.33	100.00	185.88±0.93	100.00

2.5. Fatty acid contents and compositions of Chlorella sp. UJ-3 exposed to low concentrations of Fe_3O_4 NPs

Table S3. Fatty acid contents and compositions of *Chlorella* sp. UJ-3 exposed to low concentrations of Fe_3O_4 NPs.

Fatty acids	NPs concentration (mg/L)													
	0		5		10		15		20		30		40	
	Content (mg/gDW)	Composition (%TFA)	Content (mg/gDW)	Composition (%TFA)	Content (mg/gDW)	Composition (%TFA)	Content (mg/gDW)	Composition (%TFA)	Content (mg/gDW)	Composition (%TFA)	Content (mg/gDW)	Composition (%TFA)	Content (mg/gDW)	Composition (%TFA)
C12:0	2.03±0.31	1.27±0.23	2.63±1.09	1.63±0.11	2.88±0.48	1.76±0.33	3.07±0.79	1.86±0.27	3.13±0.55	1.88±0.21	2.26±0.18	1.31±0.20	1.56±0.67	0.88±0.09
C14:0	1.22±0.08	0.76±0.02	1.09±0.08	0.68±0.25	1.10±0.10	0.67±0.05	0.86±0.06	0.52±0.02	0.89±0.17	0.53±0.01	0.96±0.03	0.56±0.05	0.85±0.05	0.48±0.01
C16:0	55.77±1.28	34.92±0.96	55.62±1.95	34.47±0.56	56.86±1.65	34.78±1.13	57.59±1.42	34.83±0.79	57.61±0.27	34.54±0.12	58.27±1.55	33.93±1.21	61.74±1.19	34.84±0.14
C16:1	3.20±0.43	2.00±0.27	2.82±0.24	1.75±0.08	2.49±0.49	1.52±0.20	3.18±0.78	1.92±0.62	3.81±0.16	2.28±0.10	3.90±0.19	2.27±0.12	3.69±0.41	2.08±0.08
C18:0	8.69±1.29	5.44±0.75	9.77±0.74	6.05±0.26	10.03±0.38	6.14±0.22	10.32±1.32	6.24±0.47	9.93±0.72	5.95±0.45	10.78±0.96	6.28±0.38	11.23±0.52	6.34±0.11
C18:1	42.12±1.41	26.37±0.72	41.79±0.44	25.90±0.27	41.88±1.23	25.62±0.56	40.28±0.97	24.36±0.55	39.85±0.35	23.89±0.23	40.06±0.45	23.32±0.21	40.19±0.43	22.68±0.14
C18:2	5.68±0.33	3.56±0.27	4.84±0.92	3.00±0.11	4.65±0.22	2.85±0.09	5.19±0.24	3.14±0.12	5.25±0.08	3.15±0.05	6.09±0.31	3.54±0.07	7.22±0.29	4.07±0.25
C18:3n6	2.68±0.07	1.68±0.02	3.48±0.29	2.16±0.14	3.47±0.53	2.12±0.26	4.02±0.34	2.43±0.15	5.41±0.84	3.24±0.13	6.31±0.37	3.68±0.29	6.76±1.04	3.82±0.46
C18:3n3	27.82±0.53	17.42±0.23	28.48±0.41	17.65±0.12	28.60±0.17	17.49±0.10	28.72±0.36	17.37±0.11	28.57±0.27	17.13±0.18	29.24±0.07	17.03±0.05	30.15±0.41	17.01±0.09
C20:0	0.32±0.02	0.20±0.02	0.36±0.05	0.22±0.02	0.39±0.01	0.24±0.01	0.38±0.03	0.23±0.02	0.40±0.03	0.24±0.01	0.54±0.07	0.32±0.02	0.38±0.02	0.21±0.01
C20:1	1.78±0.07	1.12±0.06	1.83±0.08	1.13±0.02	1.97±0.10	1.20±0.05	1.76±0.23	1.07±0.09	1.49±0.06	0.90±0.02	1.85±0.09	1.07±0.02	1.78±0.07	1.00±0.03
C20:5	7.64±0.57	4.79±0.24	7.95±0.32	4.93±0.10	8.18±0.58	5.00±0.43	9.19±0.72	5.56±0.16	9.24±0.88	5.54±0.71	10.20±0.92	5.94±0.28	10.70±0.74	6.04±0.25
C22:0	0.76±0.05	0.48±0.03	0.71±0.03	0.44±0.02	0.98±0.05	0.60±0.06	0.78±0.04	0.47±0.01	1.22±0.08	0.73±0.01	1.29±0.11	0.75±0.04	0.97±0.04	0.55±0.02
Total	159.71±3.31	100.00	161.37±1.46	100.00	163.48±2.78	100.00	165.35±1.43	100.00	166.78±3.81	100.00	171.74±2.14	100.00	177.22±3.16	100.00

2.6. Fatty acid contents and compositions of Chlorella sp. UJ-3 exposed to high concentrations of Fe_3O_4 NPs

Table S4. Fatty acid contents and compositions of *Chlorella* sp. UJ-3 exposed to high concentrations of Fe_3O_4 NPs.

Fatty acids	NPs concentration (mg/L)													
	0		60		80		100		120		140		160	
	Content (mg/gDW)	Composition (%TFA)	Content (mg/gDW)	Composition (%TFA)	Content (mg/gDW)	Composition (%TFA)	Content (mg/gDW)	Composition (%TFA)	Content (mg/gDW)	Composition (%TFA)	Content (mg/gDW)	Composition (%TFA)	Content (mg/gDW)	Composition (%TFA)
C12:0	2.15±0.21	1.38±0.05	3.62±0.14	1.91±0.07	3.00±0.10	1.41±0.06	2.73±0.32	1.16±0.09	2.09±0.15	1.00±0.06	2.12±0.10	1.07±0.07	2.22±0.17	1.16±0.06
C14:0	1.17±0.10	0.75±0.01	0.76±0.05	0.40±0.02	0.97±0.10	0.46±0.02	1.03±0.10	0.44±0.05	0.86±0.09	0.41±0.02	0.83±0.03	0.42±0.01	0.85±0.04	0.45±0.02
C16:0	50.55±1.76	32.36±0.65	61.79±1.59	32.65±0.98	70.69±0.91	33.32±1.18	77.17±2.03	32.79±1.26	71.26±2.01	34.15±1.10	67.47±1.08	34.18±0.52	64.76±1.73	33.87±0.29
C16:1	3.12±0.10	2.00±0.16	3.97±0.21	2.10±0.09	5.83±0.57	2.75±0.21	7.34±0.55	3.12±0.38	6.25±0.25	2.99±0.12	5.39±0.22	2.73±0.10	5.29±0.24	2.77±0.07
C18:0	6.04±0.22	3.87±0.12	9.89±0.57	5.22±0.23	10.77±1.14	5.08±0.84	14.54±0.50	6.18±0.12	12.44±0.50	5.96±0.30	11.87±1.00	6.01±0.82	12.42±0.38	6.49±0.32
C18:1	41.52±1.92	26.58±1.12	40.81±1.10	21.56±0.78	40.15±1.56	18.93±0.92	41.78±1.69	17.75±0.73	35.58±1.71	17.05±1.31	33.25±1.12	16.84±0.78	31.34±1.17	16.39±0.92
C18:2	7.79±0.51	4.99±0.13	8.45±0.45	4.47±0.22	9.91±1.11	4.67±0.57	12.57±0.96	5.34±0.25	9.65±0.88	4.62±0.74	8.38±0.33	4.25±0.10	8.22±0.21	4.30±0.06
C18:3n6	3.08±0.17	1.97±0.05	6.38±0.27	3.37±0.25	7.48±0.47	3.52±0.28	7.89±0.50	3.35±0.17	6.99±0.56	3.35±0.26	7.15±0.41	3.62±0.27	6.93±0.71	3.62±0.08
C18:3n3	28.77±0.65	18.42±0.24	36.80±1.55	19.44±1.19	42.18±0.88	19.88±1.18	45.48±1.51	19.33±1.13	41.18±1.50	19.73±0.54	38.34±1.91	19.42±0.73	37.43±1.97	19.58±1.05
C20:0	0.22±0.03	0.14±0.01	0.22±0.02	0.12±0.01	0.47±0.06	0.22±0.02	0.54±0.05	0.23±0.01	0.28±0.04	0.13±0.01	0.30±0.04	0.15±0.01	0.28±0.04	0.15±0.01
C20:1	0.57±0.05	0.37±0.01	1.23±0.11	0.65±0.05	1.43±0.05	0.68±0.02	1.58±0.10	0.67±0.04	1.43±0.05	0.68±0.03	1.37±0.06	0.69±0.01	1.39±0.07	0.72±0.01
C20:5	10.36±0.54	6.63±0.49	14.57±1.08	7.70±0.28	18.17±0.45	8.56±0.11	21.48±1.00	9.13±0.28	19.59±0.89	9.39±0.27	19.83±0.73	10.04±0.52	18.97±0.82	9.92±0.27
C22:0	0.87±0.05	0.56±0.02	0.76±0.05	0.40±0.02	1.09±0.08	0.52±0.01	1.20±0.06	0.51±0.02	1.10±0.10	0.53±0.03	1.12±0.09	0.57±0.02	1.12±0.10	0.59±0.01
Total	156.22±1.45	100.00	189.25±2.36	100.00	212.14±1.88	100.00	235.33±3.49	100.00	208.70±2.01	100.00	197.40±5.87	100.00	191.21±1.09	100.00

2.7. Effect of transition time on the combination strategy

Table S5. Effect of transition time on combination strategy with low-high concentration NPs treatment.

Transition time	Biomass (g/L)	Lipid production (g/L)
Day 3	0.96±0.03	0.40±0.02
Day 6	1.02±0.02	0.43±0.02
Day 9	1.08±0.02	0.45±0.01
Day 12	1.18±0.03	0.49±0.02
Day 15	1.17±0.03	0.42±0.03

Reference

[1] John, K.M.M.; Enkhtaivan, G.; Lee, J.H.; Thiruvengadam, M.; Keum, Y.S.; Kim, D.H. Spectroscopic determination of metabolic and mineral changes of soya-chunk mediated by *Aspergillus sojae*. *Food Chemistry* **2015**, 170, 1-9.

[2] Xu, L.; Guo, C.; Wang, F.; Zheng, S.; Liu, C.Z. A simple and rapid harvesting method for microalgae by in situ magnetic separation. *Bioresource Technology* **2011**, 102, 10047-10051.
